# A Questionnaire-Based Survey to Assess the Awareness and Usage of Social Media by Orthodontic Patients in Kerala, India, for Treatment-Related Information

**DOI:** 10.7759/cureus.105052

**Published:** 2026-03-11

**Authors:** Baby Jisha, Muhammed Shibin P, Shobha Sundareswaran, Prathapan Parayaruthottam, Latheef V P, Soumya Mohanan TV, Praveen S Nair

**Affiliations:** 1 Orthodontics and Dentofacial Orthopedics, Government Dental College, Kozhikode, IND; 2 Orthodontics and Dentofacial Orthopedics, Kunhitharuvai Memorial Charitable Trust (KMCT) Dental College, Kozhikode, IND; 3 Public Health Dentistry, Government Dental College, Kozhikode, IND

**Keywords:** brushing techniques, orthodontic awareness, questionnaire, social media, youtube®

## Abstract

Introduction

Social media has revolutionized healthcare information delivery, with orthodontic patients increasingly seeking treatment-related information online. This study assessed the awareness level of orthodontic patients regarding the availability and utilization of orthodontic information on social media platforms in Kerala, India.

Materials and methods

A cross-sectional survey was conducted among 400 orthodontic patients aged 15-35 years at the Government Dental College, Kozhikode, Kerala. A validated questionnaire comprising 13 questions across three domains (social media usage, access to orthodontic treatment, and orthodontic information) was administered. Content validity was assessed by a panel of orthodontists, and the questionnaire was translated into the local language.

Results

The 403 participants (55% women and 45% men) aged 17-22 years (mean: 19±4 months) primarily accessed YouTube (60.5%) and Instagram (30.5%) for orthodontic information. Chi-square (χ²) tests revealed significant associations between age and information-seeking about treatment difficulties (p=0.045) and clinic searching (p=0.002). Multiple response analysis showed a strong gender-social media preference association (χ²=25.197; p<0.05). Gender significantly influenced searching for orthodontists (p=0.026) and cost information (p=0.001). Logistic regression identified age (Exp(B)=1.091; p=0.002) and regular social media use as significant awareness predictors.

Conclusion

Orthodontic patients in Kerala actively use social media for orthodontic information, particularly YouTube and Instagram, and demonstrate willingness to follow orthodontists online, with age and usage frequency predicting information-seeking behavior despite preferring direct professional consultation for emergencies.

## Introduction

Social media refers to web-based platforms wherein users share and create content and participate in interactive discussions, sitting in different corners of the world [1]. The global social media user base has shown a consistent upward trajectory, increasing from 4.62 billion in January 2022 to 5.24 billion by February 2025, representing a 13.4% overall growth [2]. India has one of the highest numbers of social media users owing to its huge population. Kerala, one of the most literate states in India, has a high number of social media users. Millennials and Generation Z are the main contributors to social networking in India, with an average time of 5-6 hours spent daily on social media [3]. They also form the major portion of the orthodontic patient population.

The digital revolution has changed the way healthcare information is delivered across the world, especially in developing countries. It has been observed that providing audiovisual information through social media, such as YouTube, has resulted in improved care of the dentition and fixed appliances among orthodontic patients [4,5]. Studies have shown that providing information to orthodontic patients in a visual format is effective for information retention compared to other formats [6]. In recent times, clinical practitioners have their own social media handles through which they can display the types of cases done by them and the newest treatment methods and trends in orthodontics [7].

Orthodontic patients of this era are interested in newer treatment options and would like to be actively involved in the decision-making process [8]. However, unravelling the lacuna in our understanding of the extent of the usage of social media by orthodontic patients or knowing the kind of information that is sought after would help the orthodontists deliver treatment-related information effectively, which can, in turn, benefit their patients. Hence, the primary aim of this study was to assess the degree of awareness of the orthodontic patients regarding the availability and utilization of orthodontic information on social media in Kerala, India. Also, the determination of the association between demographic variables and patterns of information-seeking behavior, including platform preferences and the predictors of social media use, forms the secondary objectives of this study.

## Materials and methods

This study was a cross-sectional survey, conducted in the department of orthodontics at Government Dental College, Kozhikode, Kerala, India. Ethical approval was obtained from the Institutional Ethics Committee (IEC) of Government Dental College, Kozhikode (IEC approval number: 245/2022/DCC).

Sample size calculation

A total of 403 patients were included. Convenience sampling was undertaken, and the sample size was based on a study done by Katti and Mohan (2021) with a confidence level of 95% and an error margin of ±5% using the formula n=/z²p(1-p)/d²/, where z=1.96, p=65, and d=5 at 95% confidence [9].

Inclusion and exclusion criteria

The inclusion criteria included patients undergoing fixed orthodontic treatment in the age range of 15-35 years who are regular users of social media. The exclusion criteria included young patients with any mental or behavioral disorders affecting judgement and those who do not agree to participate or whose legal representatives do not authorize participation in the study.

Questionnaire development

A questionnaire in English was developed by a panel of six orthodontists with more than five years of clinical experience. The questionnaire comprised 13 questions across three domains pertaining to social media usage, access to orthodontic treatment, and information on orthodontic treatment (Table 1). It was translated to the local language by a team of orthodontic postgraduates proficient in both English and the local language and then back-translated.

**Table 1:** Questionnaire for assessing information-seeking in social media by orthodontic patients.

	Questions	Response	
1.	Have you started orthodontic treatment?	Yes	No
	i) If yes, approximately how long ago was that?	__________ Months	
	Do you use social media regularly?	Yes	No
	Domain 1: social media usage		
2.	Which platforms have you used to find information about brace treatment?	YouTube, Facebook, Instagram, X (Twitter), Snapchat, blogs, and others	
3.	Which is the most common social media platform that you use to gain information about braces? (tick only one)	YouTube, Facebook, Instagram, X (Twitter), Snapchat, blogs, and others	
4.	If you have used social media, did you find it useful?	Yes	No
	Domain 2: access to orthodontic treatment		
5.	Have you used social media to search for the availability of orthodontic treatment in your area?	Yes	No
6.	Have you used social media to find an orthodontic clinic?	Yes	No
7.	Have you used social media to find an orthodontist?	Yes	No
	Domain 3: information on orthodontic treatment		
8.	Have you used social media to get information on the types of braces available? Explain: __________	Yes	No
9.	Have you used social media to know about orthodontic treatment difficulties? Explain: __________	Yes	No
10.	Have you used social media to know about brushing techniques while on treatment with braces?	Yes	No
11.	Have you used social media to know about the cost of different types of braces?	Yes	No
12.	Have you used social media for managing orthodontic emergencies before contacting your doctor? Explain: __________	Yes	No
13.	If orthodontists disseminate information regarding orthodontic treatment through social media, would you use it?	Yes	No

Assessment of validity

A pilot study was done to assess face validity and to understand if the items were clear and comprehensible. Content validity was assessed carefully by a six-member panel consisting of orthodontists using the content validity index [10]. The assessment of conceptual and semantic equivalence was also done. The final version of the questionnaire was administered to patients who satisfied the inclusion criteria, and responses were recorded.

Statistical tests

Frequency distributions for responses to questionnaire items were done. Chi-square (χ²) test was done to assess the associations between gender and social media platform preferences and age groups and awareness levels. Differences in information-seeking behavior by demographic variables were also assessed using the chi-square test. Logistic regression tests were performed to find the predictors of the awareness of orthodontic content. Multiple response analysis was also carried out since subjects could select multiple responses.

## Results

**Figure 1:**
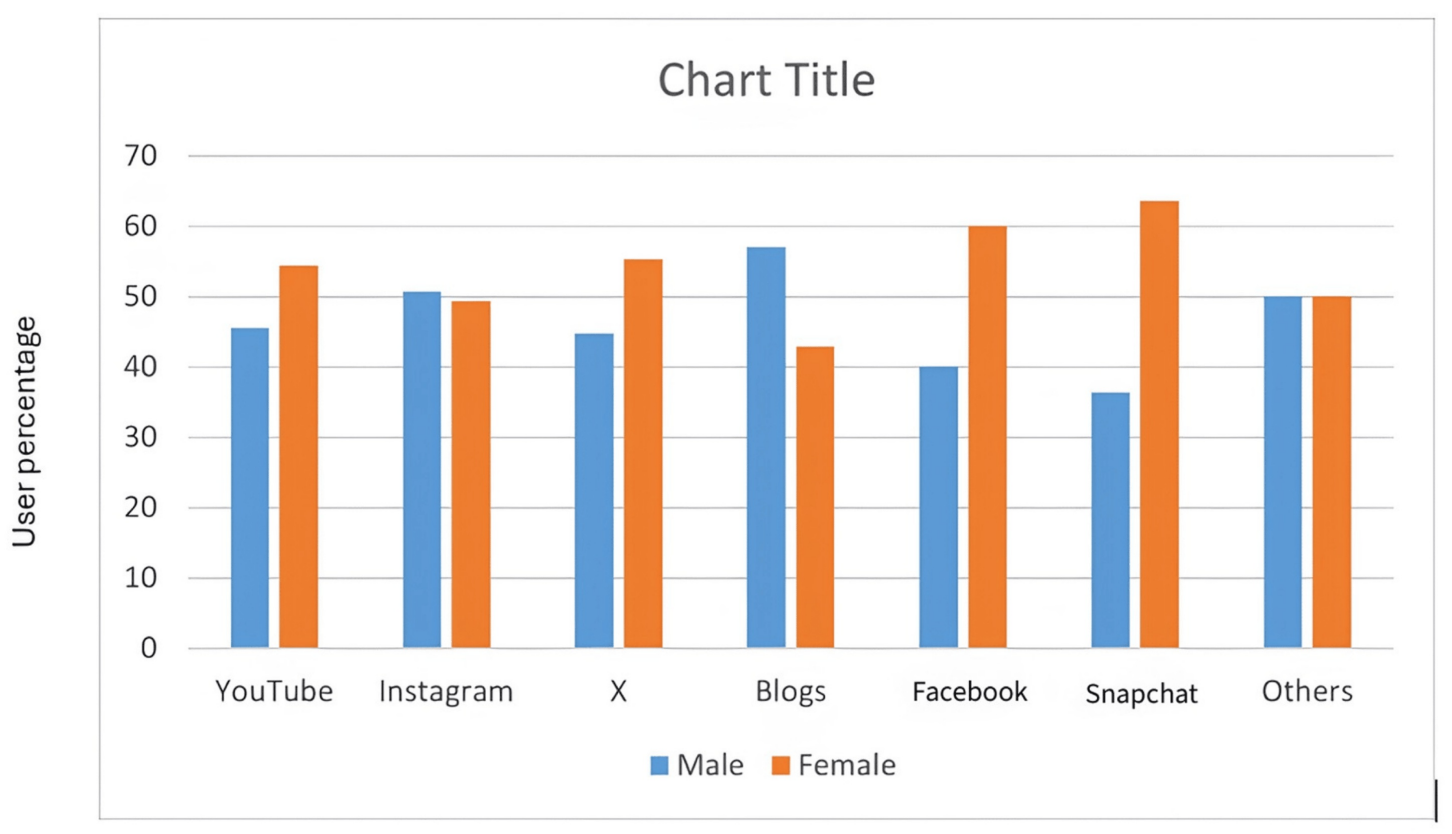
User percentage of various social media platforms based on gender.

The responses recorded from the participants were analyzed by descriptive statistics using version 24 of the Statistical Package for Social Sciences (SPSS) software (IBM Corp., Armonk, NY). The participants’ ages ranged from 17 to 22 years, with an average age of 19 years plus four months. Out of the 403 patients who responded, 206 (56.1%) were women, and 161 (43.9%) were men (Table 2). The most popular social media platforms accessed to obtain information were YouTube (n=222, 60.5%) and Instagram (n=112, 30.5%), out of which a higher proportion of women (n=143, 69.4%) use the former, compared to men (n=79, 49.1%) (Figure 1). Chi-square test assessed the association between gender and social media preferences, and the results showed a strong association (χ²=25.197; p<0.05) (Table 3). Chi-square test also revealed that “information-seeking about orthodontic treatment difficulties” (p=0.045) and “searching for an orthodontic clinic” (p=0.002) showed statistically significant association with age (Table 4).

**Table 2:** Demographic variables: age and gender distribution across sample (number and percentage).

Age (in years)		Gender	
Below 18	178 (48.5%)	Male	161 (43.9%)
Above 18	189 (51.5%)	Female	206 (56.1%)

**Table 3:** Association between gender and social media platform preferences. Chi-square test is done. Observations are considered statistically significant at p<0.05. Values are expressed as a percentage of the sample size.

Social media platform	Gender		Chi-square	P-value
	Male, n (%)	Female, n (%)		
YouTube	79 (49.1)	143 (69.4)	25.197	0.001
Instagram	62 (38.5)	50 (24.3)		
X	4 (2.5)	6 (2.9)		
Blogs	16 (9.9)	4 (1.9)		
Facebook	0 (0)	2 (1)		
Snapchat	0 (0)	1 (0.5)		

**Table 4:** Association between age and information-seeking. Chi-square test is done to determine the association between age and information-seeking (significant: p<0.05). Values are expressed as a percentage of the sample size.

Information search variables		Age (grouped)		Chi-square	P-value
		Below 18, n (%)	18-30, n (%)		
Information on types of braces	No	83 (46.9)	99 (52.4)	1.101	0.298
	Yes	94 (53.1)	90 (47.6)		
Information on treatment difficulties	No	109 (61.6)	96 (50.8)	4.318	0.045
	Yes	68 (38.4)	93 (49.2)		
Information on brushing techniques	No	80 (45.2)	95 (50.3)	0.940	0.348
	Yes	97 (54.8)	94 (49.7)		
Information on the cost of braces	No	122 (68.9)	122 (64.6)	0.788	0.437
	Yes	55 (31.1)	67 (35.4)		
Searching for orthodontic treatment availability	No	168 (94.9)	178 (94.2)	0.096	0.821
	Yes	9 (5.1)	11 (5.8)		
Searching for an orthodontic clinic	No	147 (83.1)	130 (68.8)	10.110	0.002
	Yes	30 (16.9)	59 (31.2)		
Searching for an orthodontist	No	167 (94.4)	167 (88.4)	4.111	0.063
	Yes	10 (5.6)	22 (11.6)		

Eighty-eight percent (n=354) of the subjects found information on orthodontics useful, while the rest (12%, n=48) did not find it helpful. Statistically significant associations between gender and information-seeking were observed on two variables, i.e., searching for an orthodontist (p=0.026) and information on the cost of braces (p=0.001), with dominance of the female gender (Table 5).

**Table 5:** Association between gender and information-seeking. Chi-square test is done to determine the association between gender and information-seeking behavior (significant: p<0.05). Values are expressed as a percentage of the sample size.

Information search variable		Gender		Chi-square	P-value
		Male, n (%)	Female, n (%)		
Searching for orthodontic treatment availability	No	153 (95)	193 (94.1)	0.137	0.819
	Yes	8 (5)	12 (5.9)		
Searching for an orthodontic clinic	No	130 (80.7)	147 (71.7)	4.003	0.050
	Yes	31 (19.3)	58 (28.3)		
Searching for an orthodontist	No	153 (95)	181 (88.3)	5.132	0.026
	Yes	8 (5)	24 (11.7)		
Information on types of braces	No	79 (49.1)	103 (50.2)	0.05	0.834
	Yes	82 (50.9)	102 (49.8)		
Information on treatment difficulties	No	97 (60.2)	108 (52.7)	2.095	0.168
	Yes	64 (39.8)	97 (47.3)		
Information on brushing techniques	No	81 (50.3)	94 (45.9)	0.718	0.402
	Yes	80 (49.7)	111 (54.1)		
Information on the cost of braces	No	92 (57.1)	152 (74.1)	11.732	0.001
	Yes	69 (42.9)	53 (25.9)		
The use of social media for emergency management	No	156 (96.9)	182 (88.8)	8.403	0.005
	Yes	5 (3.1)	23 (11.2)		

Ninety-five percent (n=382) of the patients preferred to contact their orthodontist during emergency situations, but a very small fraction of patients tried to resolve it with the help of social media. A significant association was found between gender and willingness to follow orthodontists online, with women being more willing compared to men (Table 6). Logistic regression was done to find the specific variables that predict awareness, out of which age (Exp(B)=1.091; p=0.002) and the regular use of social media (B=19.876) were found to be the best predictors.

**Table 6:** Association between gender and willingness to follow orthodontists online. Chi-square test is done to evaluate the association between gender and willingness to follow orthodontists online (significant: p<0.05). Values are expressed as a percentage of the sample size.

Variable		Gender		Chi-square	P-value
		Male, n (%)	Female, n (%)		
Willingness to use orthodontist content	No	2 (1.2)	12 (6)	5.375	0.026
	Yes	159 (98.8)	189 (94)		

## Discussion

The present study demonstrates that there is good awareness among the population of Kerala of the presence of information related to orthodontics on social media. The majority of the surveyed patients were aware of this, which is higher than that observed in a similar study by D’cunha et al. [11]. Since social media is chiefly used for personal purposes, such as keeping in touch with family and friends, by the common man, orthodontic information comes to their attention only when they search actively and when there is adequate marketing as well [12]. Age was found to be significantly associated with seeking “information on treatment difficulties,” with young adults (18-30) being more concerned. Similar findings were observed in a study by Kim, the probable reasons for this being the above age group having a mature dentition with multiple restorations, periodontal problems, and probably the apprehension about treatment difficulties interfering with personal and professional life [13].

A higher percentage of adult patients (31.2%, n=126) searched for an orthodontic clinic, which was statistically significant and also slightly less than that found in a study by Abdulrahman et al. (47.17%) [14]. Eighty-eight percent of the subjects found social media useful for obtaining knowledge on various aspects of orthodontic treatment, whereas the remaining did not find it so, the reasons being the inability to make a decision based on the available online content and apprehension over the credibility of the same. This figure is more than that found in a study by Crispino et al. (64%-68%) [15].

According to this study, the most used social media platform to obtain information about braces was found to be YouTube, which is also among the top three widely used ones across the world [16]. YouTube was followed by Instagram and blog posts, respectively. Since these platforms focus primarily on audiovisual content of shorter duration, patients would find it easier to assimilate and interpret the information delivered in an effective manner. As reported by Tamošiūnaitė et al., most of the content being posted on YouTube and Instagram is not by dental professionals, i.e., dentists or orthodontists [17]. Hence, the educational and scientific quality of the videos or pictures can be questionable or possibly misleading to the general population.

About one-fourth (n=100, 24%) of the surveyed patients had searched for an orthodontic clinic in and around their residence through social media, and an even smaller percentage (n=20, 5%) had searched for a specific orthodontist. A majority had reported that they had located the institution through dentists or pedodontists’ referral or those from peers and relatives. This finding is similar to the results of studies done by Katti et al. [9] and Kothari et al. [18]. It can be inferred that patients in Kerala continue to depend on word-of-mouth recommendations from family and friends rather than online sources when choosing an orthodontist, aligning with the findings of Adanan et al. [19]. As reported by Edwards et al., the chief factor in the selection of an orthodontist is his/her reputation [8]. Nelson et al.’s study also revealed that orthodontists with a strong online presence through practice websites and social media platforms attracted more new patients than those relying on newspaper advertisements for marketing [20]. This becomes relevant in that most patients were interested to follow a social media portal offered by their own orthodontist. It can also be assumed that patients place greater trust in the information provided by their orthodontist compared to other sources. A strong online presence with scientifically sound videos can boost an orthodontist’s professional image and, in turn, help them attract new patients [21].

About 51% (n=205) of the subjects have searched for different types of braces available, including aligners. Patient comments on this question indicate that aligners were the most sought-after treatment modality. A study by Yavan and Gökçe also reported that aligners were the most shared modality, whereas functional/orthopedic appliances and orthognathic surgical procedures were the least shared treatment options on social media, which backs the above findings [22]. Although ample information on treatment methods such as functional and orthopedic appliances and orthognathic surgery domains is available online, the fact that these are explored less compared to aligners indicates an interest toward the aesthetic treatment opportunity provided by aligners. However, the absence of standardized regulation for online information presents challenges for orthodontists in managing patients who have been influenced by potentially misleading content [23].

A small fraction of the patients (33%, n=133) checked the cost of undergoing an orthodontic treatment on social media, indicating that cost is not an important factor in decision-making. However, patients who searched for cost also reported that they did not get any useful information about this topic. Brushing techniques during orthodontic treatment were sought by 52% (n=210) of the participants, with YouTube being the most frequently accessed platform for this information. A study by Scribante et al. [4] found that short videos on brushing with written instructions on Instagram, along with verbal instructions, significantly improved oral hygiene among patients with fixed appliances [24]. Therefore, orthodontists can recommend YouTube or Instagram videos to patients after systematically evaluating the quality and credibility of the content. In response to the question on the management of orthodontic emergencies by themselves with the help of social media, 95% (n=382) of the subjects stated that they contacted their orthodontist through calls or via smartphone applications such as WhatsApp. Only 5% (n=20) tried to solve the issues by looking up on social media sites such as YouTube. The common problems that patients apparently tried to solve by themselves were with respect to protruding ends of archwires and the breakage of brackets. Previous studies had reported similar findings, and this tendency of patients can be attributed to the COVID-19 pandemic, wherein orthodontic practice was limited during its peak and patients resorted to online platforms for information [24,25].

Although this study has tried to cover most of the aspects of the social media awareness of orthodontic content in Kerala, an in-depth understanding of what they seek through social media use is required. One of the limitations of this study is the lack of assessment of factors such as education and socio-economic status of the subjects, which would have helped in the better understanding of predictive factors of information-seeking behavior. These factors can greatly help the orthodontists to provide scientific information on orthodontic treatment, which can be utilized by their patients, as well as the whole patient population, to clear their apprehensions, which can in turn lead to the successful completion of treatment.

Future research should evaluate content quality across platforms updated on a regular basis, assess the impact of practitioner-created materials on treatment outcomes, and explore the optimal integration of digital resources, along with traditional clinical communication, to enhance patient education and treatment compliance.

## Conclusions

YouTube is the most frequently used platform by orthodontic patients, followed by Instagram. Contents on different types of braces, brushing techniques, and treatment difficulties, such as pain and ulceration, were searched by patients in these platforms.

The age of subjects and regular use of social media were found to be the predictive factors for information-seeking behavior. The significant willingness to follow orthodontists’ social media channels presents an untapped opportunity for providing credible, evidence-based information.
